# Silica Nanoparticle-Reinforced Bioactive Oxidized Alginate/Polyacrylamide–Gelatin Interpenetrating Polymer Network Composite Hydrogels

**DOI:** 10.3390/gels11090748

**Published:** 2025-09-17

**Authors:** Yanan Bu, Jiayi Liu, Jiji Fan, Xiuqiong Chen, Huiqiong Yan, Qiang Lin

**Affiliations:** 1 Key Laboratory of Tropical Medicinal Resource Chemistry of Ministry of Education, Key Laboratory of Tropical Medicinal Plant Chemistry of Hainan Province, College of Chemistry and Chemical Engineering, Hainan Normal University, Haikou 571158, China; buynedu@163.com (Y.B.); ljy151930@163.com (J.L.); fjj17785390542@163.com (J.F.); chenxiuqiongedu@163.com (X.C.); linqianggroup@163.com (Q.L.); 2 Key Laboratory of Water Pollution Treatment & Resource Reuse of Hainan Province, College of Chemistry and Chemical Engineering, Hainan Normal University, Haikou 571158, China; 3 Key Laboratory of Natural Polymer Functional Material of Haikou City, College of Chemistry and Chemical Engineering, Hainan Normal University, Haikou 571158, China; 4 Tropical Functional Polymer Materials Engineering Research Center of Hainan, College of Chemistry and Chemical Engineering, Hainan Normal University, Haikou 571158, China

**Keywords:** oxidized alginate/polyacrylamide matrices, silica nanoparticle, interpenetrating polymer network, composite hydrogels, biological properties

## Abstract

Alginate hydrogels are promising tissue engineering biomaterials due to their biocompatibility and structural similarity to the extracellular matrix, but their poor mechanical strength, rapid degradation, and lack of bioactivity limit applications. To address this, a novel oxidized alginate/polyacrylamide/silica nanoparticle–gelatin (OA/PAAm/SiO_2_-GT) composite hydrogel was developed using an interpenetrating polymer network (IPN) strategy, reinforced with silica nanoparticles and coated with gelatin. The influence of SiO_2_ content on the microstructure, mechanical properties, swelling behavior, biodegradability, biomineralization, and cytocompatibility of the composite hydrogel was systematically investigated. Experimental results revealed that SiO_2_ nanoparticles interacted with the polymer matrix within the composite hydrogel. With increasing content of SiO_2_, the porosity of the OA/PAAm/SiO_2_-GT composite hydrogel gradually decreased, while the mechanical properties exhibited a trend of initial enhancement followed by reduction, with maximum compressive strength at a SiO_2_ content of 1.0% (*w*/*v*). Moreover, the incorporation of SiO_2_ nanoparticles effectively modulated the swelling behavior, biodegradability, and biomineralization capacity of the composite hydrogel under in vitro conditions. Meanwhile, the OA/PAAm/SiO_2_-GT composite hydrogel supported favorable cell adhesion and proliferation, optimal at a SiO_2_ content of 0.5% (*w*/*v*). Furthermore, with increasing concentration of SiO_2_ nanoparticles, the intracellular alkaline phosphatase (ALP) activity progressively increased, suggesting a promotive effect of SiO_2_ nanoparticles on the osteogenic differentiation of MG63 cells. Therefore, the incorporation of SiO_2_ nanoparticles into the OA/PAAm IPN matrices provides an effective means to tailor its biological properties, rendering it great potential for biomedical applications such as tissue engineering.

## 1. Introduction

Hydrogels are three-dimensional polymeric networks that retain a large amount of water. Due to their similarity to the native extracellular matrix, hydrogels are widely used in drug delivery, tissue engineering scaffolds, and wound dressings [1,2]. However, the biomedical utility of conventional hydrogels is often limited by their weak mechanical properties, which fail to adequately replicate the mechanical microenvironment essential for cellular activities [3]. In contrast, interpenetrating polymer network (IPN) hydrogels have garnered significant research interest due to their enhanced mechanical performance [4]. An IPN is defined as a combination of two or more independently crosslinked networks that are physically interwoven and interpenetrating, while each remains chemically distinct [5]. In a typical IPN structure, the first network is often made of natural polymers such as sodium alginate, hyaluronic acid, or chitosan, providing high stiffness. The second network, usually composed of synthetic polymers like polyacrylamide, contributes superior ductility. The synergistic interaction between these networks results in composite hydrogels exhibiting both high strength and excellent toughness [6,7].

Alginate is a linear water-soluble polysaccharide composed of (1-4)-linked *β*-D-mannuronic acid (M blocks) and (1-4)-linked *α*-L-guluronic acid (G blocks), which form homopolymeric (MM or GG) and heteropolymeric sequences (MG or GM) [8,9]. Owing to its compositional similarity to the natural extracellular matrix, alginate-based hydrogels can provide a favorable cellular microenvironment that supports and modulates critical cell behaviors such as adhesion, proliferation, migration, and differentiation [10,11]. Furthermore, alginate is widely available, exhibits mild gelation behavior, and is non-toxic, making it extensively applicable in medical fields including tissue engineering and wound dressing [12,13]. Although widely studied for biomedical hydrogels, alginate hydrogels have inherent limitations, including low compressive strength, lack of mammalian cell adhesion sites, and slow, uncontrolled degradation, which limit their wider use [14,15]. To address these functional shortcomings, researchers often incorporate two or more materials to form composite hydrogels with improved load-bearing capacity. The introduction of various reinforcing agents not only enhances the mechanical properties of these hybrid or composite hydrogels but can also impart additional unique and desirable functionalities to the material system [16,17].

Silica (SiO_2_) nanoparticles, as the primary constituent of bioactive glass, exhibit outstanding mechanical strength, thermal stability, and biocompatibility [18]. Owing to its high specific surface area and favorable biocompatibility, SiO_2_ is widely utilized in biomedical applications such as tissue engineering scaffolds, drug delivery systems, and wound dressings [19,20]. Studies have indicated that SiO_2_ nanoparticles demonstrate significant bioactivity in bone repair materials by suppressing osteoclastogenesis and stimulating osteogenic differentiation [21]. As a reinforcing phase, SiO_2_ nanoparticles effectively enhance both the physicochemical properties and biological functions of composite hydrogels. The high specific surface area considerably increases the exposure of hydrophilic silanol groups (Si–OH). These groups can form hydrogen bonds with functional groups in the polymer matrix, resulting in strong interfacial interactions and improved mechanical strength of the composite hydrogels [14]. Furthermore, Si–OH groups not only facilitate the adhesion and proliferation of osteoblasts but also serve as active sites for biomimetic mineralization, guiding the nucleation and growth of bone-like apatite [22,23,24,25].

**Scheme 1:**
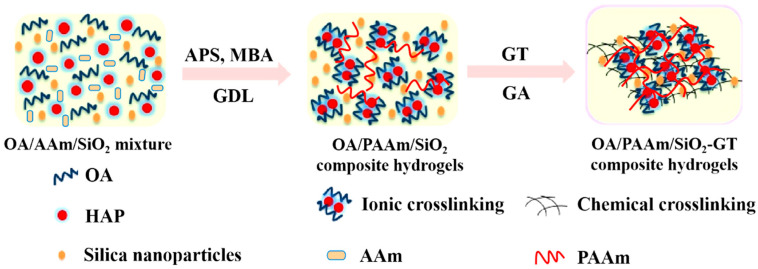
Schematic illustration of the fabrication of OA/PAAm/SiO_2_-GT composite hydrogels through a combination of IPN technology, SiO_2_ nanoparticle reinforcement, and surface coverage with GT.

Furthermore, oxidized alginate hydrogels have attracted research interest as biodegradable materials for tissue engineering applications. Compared with native alginate, partially oxidized alginate (OA) exhibits a higher degradation rate and a greater number of reactive functional groups [26]. It is reported that OA/gelatin hybrids are among the most widely employed systems in osteoarthritis research [27]. The amino groups in gelatin can react with the aldehyde groups in OA via Schiff base formation. This reaction increases crosslinking density and, as a result, improves the mechanical strength of the composite hydrogel. Additionally, coating the composite hydrogel with a gelatin solution can further enhance its cell adhesion properties. Gelatin exposes RGD peptide sequences on the surface, which specifically bind to integrin receptors on cells, thereby promoting adhesion and enhancing the overall bioactivity of the composite hydrogels [28,29,30]. Building upon these foundations, the key innovation of this work lies in the integrated design and fabrication of an oxidized alginate/polyacrylamide/silica nanoparticle–gelatin (OA/PAAm/SiO_2_-GT) composite hydrogel. This approach synergistically combined IPN technology, silica nanoparticle reinforcement, and surface coverage with gelatin, as illustrated in Scheme 1, to comprehensively address the persistent limitations of conventional alginate hydrogels, such as inadequate mechanical strength, uncontrolled degradation, and lack of bioactivity. To the best of our knowledge, this represents a novel strategy that systematically tackles multiple functional deficiencies through a multi-component and multi-scale design. The structure and properties of the resulting composite hydrogels were thoroughly investigated as a function of SiO_2_ nanoparticle content using Fourier transform infrared spectroscopy (FT-IR), thermogravimetric analyses (TGA) and X-ray diffraction (XRD), swelling tests, biodegradation assays, biomineralization studies, and cytocompatibility evaluations that were foundational for future in vivo applications. This work provides a holistic material solution aimed at enhancing the applicability of alginate-based hydrogels in demanding biomedical contexts.

## 2. Results and Discussion

### 2.1. Interactions Among the Components of Composite Hydrogels

**Figure 1:**
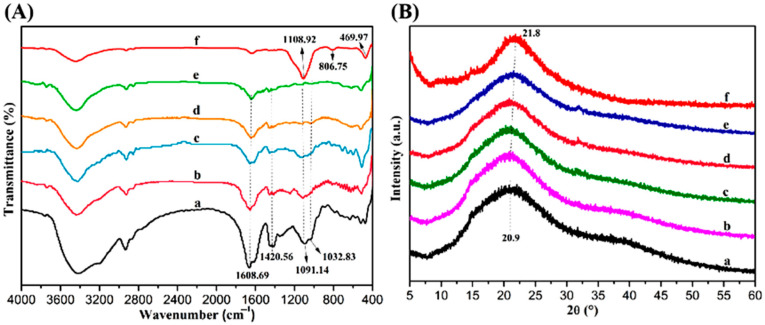
(**A**) FT-IR spectra; (**B**) X-ray diffractions of the composite hydrogels: (a) OA/PAAm-GT, (b) OA/PAAm/0.5%SiO_2_-GT, (c) OA/PAAm/1.0%SiO_2_-GT, (d) OA/PAAm/1.5%SiO_2_-GT, (e) OA/PAAm/2.0%SiO_2_-GT, and (f) SiO_2_ nanoparticles.

The interactions among the components of the OA/PAAm/SiO_2_-GT composite hydrogel were investigated using FT-IR, XRD, and TGA. Figure 1A displays the FT-IR spectra of OA/PAAm-GT, OA/PAAm/SiO_2_-GT composite hydrogels, and SiO_2_ nanoparticles. The absorption peaks of OA/PAAm-GT composite hydrogels at 1608.69, 1420.56, 1091.14, and 1032.83 cm^−1^ were attributed to the asymmetric and symmetric stretching vibrations of -COO^−^ on the OA molecular chain and the elastic vibration of C–O–C on the sugar backbone, respectively [31,32]. The characteristic peaks of SiO_2_ nanoparticles at 1108.92, 806.75, and 469.97 cm^−1^ were ascribed to the asymmetric stretching vibration of Si–O–Si and the symmetric stretching and bending vibrations of Si–O, respectively [33]. A comparison revealed that the FT-IR spectra of OA/PAAm-GT and OA/PAAm/SiO_2_-GT composite hydrogels exhibited similar peak positions. However, compared with the OA/PAAm-GT composite hydrogels, the -OH stretching vibration peak at 3420.22 cm^−1^ for OA/PAAm/SiO_2_-GT composite hydrogels with different SiO_2_ contents showed a blue shift and decreased intensity, while the intensity of the absorption peak at 469.97 cm^−1^ increased. This phenomenon was attributed to the incorporation of SiO_2_ nanoparticles into the polymer matrices, which reduced the intramolecular hydrogen bonding of OA and enhanced the intermolecular hydrogen bonding interactions among OA, SiO_2_ nanoparticles, and PAAm [34]. These results indicated that the SiO_2_ nanoparticles were well dispersed within the polymer matrices, leading to interfacial interactions [28].

Figure 1B presents the XRD patterns of OA/PAAm-GT, OA/PAAm/SiO_2_-GT composite hydrogels, and SiO_2_ nanoparticles. As observed, the OA/PAAm-GT composite hydrogels exhibited a broad diffraction peak at 2*θ* = 20.9°, corresponding to its hydrated crystal diffraction peak [9,11]. In contrast, the SiO_2_ nanoparticles displayed a broad single peak at 2*θ* = 21.8° assigned to the (101) crystal plane, consistent with the standard spectrum of SiO_2_ (JCPDS #77-0605), indicating that the SiO_2_ nanoparticles possessed a cristobalite crystal structure [14]. With increasing SiO_2_ content, the peak intensity at 2*θ* = 21.8° in the OA/PAAm/SiO_2_-GT composite hydrogels gradually increased, suggesting that the SiO_2_ nanoparticles were effectively dispersed within the polymer matrices, resulting in new interactions [33].

**Figure 2:**
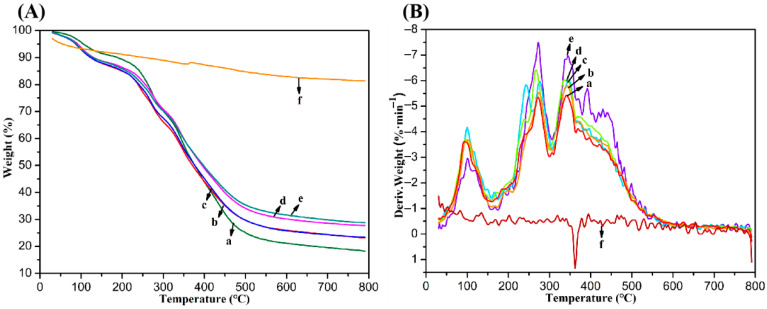
(**A**) TGA curves; (**B**) DTG curves of the composite hydrogels: (a) OA/PAAm-GT, (b) OA/PAAm/0.5%SiO_2_-GT, (c) OA/PAAm/1.0%SiO_2_-GT, (d) OA/PAAm/1.5%SiO_2_-GT, (e) OA/PAAm/2.0%SiO_2_-GT, and (f) SiO_2_ nanoparticles.

Thermogravimetric analysis (TGA) serves as a powerful tool for evaluating the thermal stability of samples and can be utilized to analyze their structural characteristics. The weight of a sample changes in response to temperature variations, and the thermal stability of a material can be assessed by comparing its final weight loss percentage. Figure 2A presents the TGA curves of OA/PAAm-GT, OA/PAAm/SiO_2_-GT composite hydrogels, and SiO_2_ nanoparticles. As seen, the OA/PAAm/SiO_2_-GT composite hydrogels exhibited superior thermal stability compared to the OA/PAAm-GT composite hydrogels, which could be attributed to interactions between the polymer chains and SiO_2_ nanoparticles [28]. SiO_2_ was known for its excellent thermal barrier properties [14], demonstrating a weight loss of only 18.63% at 800 °C. In the OA/PAAm/SiO_2_-GT composite hydrogel, the weight loss percentage gradually decreased with increasing SiO_2_ content, ranging from 81.65 to 71.20%. This indicated that the incorporation of SiO_2_ enhanced the thermal stability of the composite hydrogels to a certain extent. Figure 2B displays the derivative thermogravimetry (DTG) curves of OA/PAAm-GT, OA/PAAm/SiO_2_-GT composite hydrogels, and SiO_2_ nanoparticles. It can be observed that the weight loss process for all materials primarily occurred in three stages. The first stage, around 100 °C, was mainly due to the removal of physically adsorbed water [35]. The second stage, within the range of 200–300 °C, showed the most rapid weight loss, primarily resulting from the thermal decomposition of OA and GT, along with partial decomposition of PAAm [36,37]. The third stage, between 300–500 °C, was predominantly associated with further thermal decomposition of PAAm [5]. The OA/PAAm/SiO_2_-GT composite hydrogels exhibited a higher initial decomposition temperature than OA/PAAm-GT hydrogels, suggesting that interactions between SiO_2_ nanoparticles and the polymer matrix improved the thermal stability of the composite hydrogels [38].

### 2.2. Morphological Characteristics and Mechanical Properties of Composite Hydrogels

**Figure 3:**
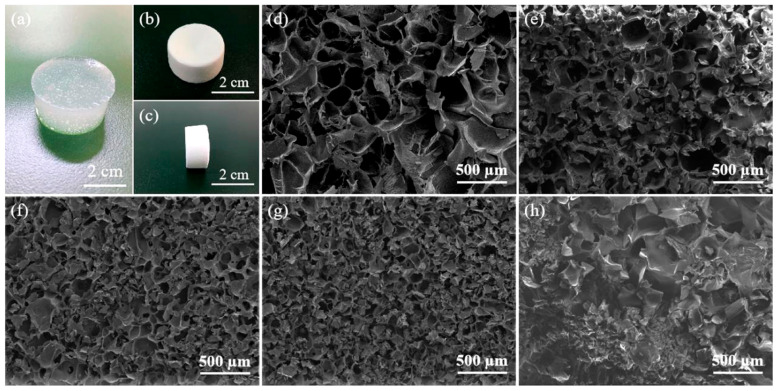
(**a**–**c**) Physical photographs of OA/PAAm/SiO_2_-GT composite hydrogels; SEM images of pore structures for the composite hydrogels: (**d**) OA/PAAm-GT; (**e**) OA/PAAm/0.5%SiO_2_-GT; (**f**) OA/PAAm/1.0%SiO_2_-GT; (**g**) OA/PAAm/1.5%SiO_2_-GT; and (**h**) OA/PAAm/2.0%SiO_2_-GT.

The pore structure morphology and mechanical properties of the composite hydrogels were examined using scanning electron microscopy (SEM) and a universal material testing machine. Figure 3 shows the pore morphology of the composite hydrogels after freeze-drying. It was found that all composite hydrogels fabricated via interpenetrating polymer network (IPN) technology exhibited regular three-dimensional (3D) architectures with porous structures. It is reported that the pore structure of composite hydrogels is closely related to biological characteristics such as cell proliferation, since an appropriate pore size not only supports cell growth but also facilitates the transport of metabolic waste and nutrients [11]. However, due to variations in the amount of SiO_2_ added in the OA/PAAm/SiO_2_-GT composite hydrogels, differences in pore density and size were evident. The OA/PAAm-GT composite hydrogels without SiO_2_ exhibited larger pores and relatively sparse distributions compared to the OA/PAAm/SiO_2_-GT composite hydrogels. In contrast, the OA/PAAm/SiO_2_-GT hydrogels revealed a denser pore structure, significantly reduced pore size, and a more orderly pore arrangement. The corresponding porosity measurements were summarized in Table 1. This phenomenon could be attributed to the homogeneous dispersion of SiO_2_ nanoparticles within the polymer matrices of the composite hydrogels. The strong interfacial interactions provided physical support to the pore walls, resulting in reduced overall porosity and the formation of a more regular and compact pore structure [39,40]. The improved structural regularity also contributed to enhanced mechanical properties [14]. These results indicated that the SiO_2_ content influenced the pore morphology of OA/PAAm/SiO_2_-GT composite hydrogels. Therefore, adding SiO_2_ nanoparticles could modulate the physicochemical properties by altering the pore architecture [15].

**Figure 4:**
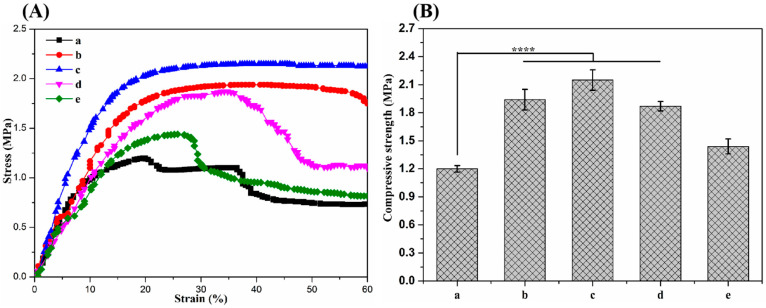
(**A**) Stress–strain curves; (**B**) compressive strength of composite hydrogels: (a) OA/PAAm-GT, (b) OA/PAAm/0.5%SiO_2_-GT, (c) OA/PAAm/1.0%SiO_2_-GT, (d) OA/PAAm/1.5%SiO_2_-GT, and (e) OA/PAAm/2.0%SiO_2_-GT (error bars represent the standard deviation of five replicates). **** represents *p* < 0.01, indicating that the difference is statistically significant.

The pore structure of the composite hydrogels directly affects their mechanical properties, which are critical for maintaining structural integrity in vivo and withstanding physiological loads [41]. Figure 4A showed the stress–strain curves of the alginate composite hydrogels. The highest linear point of each curve was the compressive strength of the composite hydrogel. Figure 4B presents the compressive strength of OA/PAAm-GT and OA/PAAm/SiO_2_-GT composite hydrogels. The compressive strength of OA/PAAm/SiO_2_-GT composite hydrogels was significantly higher than that of OA/PAAm-GT composite hydrogels. This enhancement was due to the incorporation of SiO_2_ nanoparticles into the OA/PAAm IPN matrices, which led to a more compact network structure and interactive forces between the nanoparticles and polymer chains, thereby improving mechanical performance [42]. However, as the SiO_2_ content increased, the compressive strength of the OA/PAAm/SiO_2_-GT composite hydrogels first increased and then decreased. The optimal compressive strength of 2.15 MPa was achieved at a SiO_2_ concentration of 1.0% (*w*/*v*). Although this value remains modest compared to native cortical bone (typically in the range of 130–180 MPa), it falls within the acceptable range for certain non-load-bearing bone tissue engineering applications and is superior to many previously reported alginate–gelatin-based hydrogels [27,34]. This result indicated that continuously increasing the SiO_2_ content did not indefinitely improve mechanical properties. Excessive SiO_2_ nanoparticles tended to aggregate within the polymer matrices, leading to inhomogeneous dispersion. After freeze-drying, these poorly dispersed nanoparticles often remained on the surface of composite hydrogels in powder form, thereby compromising the mechanical properties of the composite hydrogels [40].

### 2.3. Swelling and Biodegradation Properties of Composite Hydrogels

**Figure 5:**
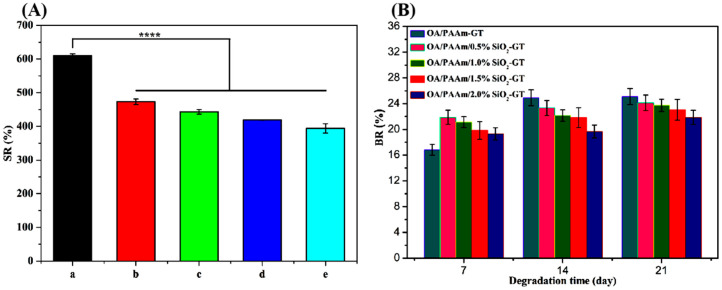
(**A**) Swelling ratio (SR); (**B**) biodegradation rate (BR) of the composite hydrogels: (a) OA/PAAm-GT, (b) OA/PAAm/0.5%SiO_2_-GT, (c) OA/PAAm/1.0%SiO_2_-GT, (d) OA/PAAm/1.5%SiO_2_-GT, and (e) OA/PAAm/2.0%SiO_2_-GT (error bars represent the standard deviation of three replicates). **** represents *p* < 0.01, indicating that the difference is statistically significant.

The in vitro swelling behavior of the composite hydrogels reflects their ability to absorb culture medium under physiological conditions [25]. Appropriate swelling performance not only helps maintain the mechanical integrity of the composite hydrogels but also facilitates the transport of nutrients and metabolic waste, thereby promoting cell adhesion and infiltration [39]. Figure 5A illustrates the swelling ratio (SR) of OA/PAAm-GT and OA/PAAm/SiO_2_-GT composite hydrogels. Among all the samples, OA/PAAm-GT exhibited the highest SR, reaching 609.50 ± 5.92%. The incorporation of SiO_2_ nanoparticles significantly reduced the SR, which decreased progressively with increasing SiO_2_ content. This effect is mainly due to the excellent colloidal properties of SiO_2_, which allow it to disperse uniformly within the OA/PAAm IPN matrices. As a result, the composite hydrogel structure became more compact, which impeded the infiltration of water molecules to a certain extent [33,38]. Figure 5B presents the biodegradation rates (BR) of OA/PAAm-GT and OA/PAAm/SiO_2_-GT composite hydrogels. It can be observed that the BR for the composite hydrogels increased gradually over time. The BR of SiO_2_-incorporated OA/PAAm/SiO_2_-GT composite hydrogels was lower than that of the OA/PAAm-GT composite hydrogels. This was likely due to the high specific surface area of SiO_2_ nanoparticles, which promoted the formation of a dense interfacial structure with the polymer matrices, thereby enhancing resistance to degradation [18]. Furthermore, the BR of OA/PAAm/SiO_2_-GT composite hydrogels decreased as the SiO_2_ content increased. These results suggest that stronger interfacial interactions between the SiO_2_ nanoparticles and the OA/PAAm IPN matrices led to a more tightly crosslinked network, thereby reducing biodegradability [43]. Therefore, adjusting the SiO_2_ content provided an effective means to modulate both the swelling and biodegradation properties of the composite hydrogels.

### 2.4. Biomineralization Study of Composite Hydrogels

**Figure 6:**
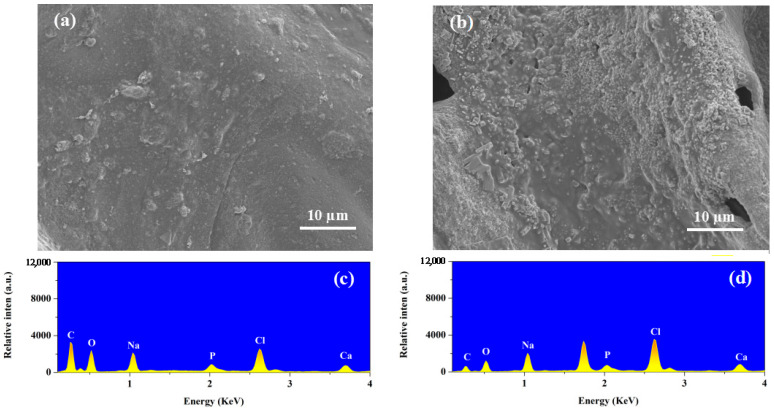
SEM images of (**a**) OA/PAAm-GT composite hydrogels; (**b**) OA/PAAm/1.0%SiO_2_-GT composite hydrogels; EDX spectra of apatite layers generated from (**c**) OA/PAAm-GT composite hydrogels and (**d**) OA/PAAm/1.0%SiO_2_-GT composite hydrogels.

The in vitro biomineralization capacity of the OA/PAAm/SiO_2_-GT composite hydrogels was evaluated by immersing them in simulated body fluid (SBF) for 14 days. As shown in Figure 6a,b, after 14 days of incubation at 37 °C, both OA/PAAm-GT and OA/PAAm/SiO_2_-GT composite hydrogels exhibited the formation of apatite mineralization layers on their surfaces. However, the OA/PAAm/SiO_2_-GT composite hydrogels displayed a more pronounced and dense layer of apatite particles compared to OA/PAAm-GT composite hydrogels, indicating that the incorporation of SiO_2_ nanoparticles effectively enhanced biomineralization [14]. During the mineralization process, the carboxyl groups on the surface of the OA/PAAm/SiO_2_-GT composite hydrogels act as nucleation sites for alginate. Additionally, the incorporation of SiO_2_ introduces further nucleation sites that promote the formation of calcium phosphate clusters. Following nucleation, hydroxyapatite crystal growth proceeds via the initial formation of Ca-rich amorphous calcium phosphate (ACP), which interacts with anions in the SBF to form Ca-poor ACP, a known precursor to crystalline apatite. Subsequent ion incorporation, including calcium, phosphate, sodium, magnesium, and carbonate, enables the apatite to develop structural and compositional features resembling those of natural bone mineral [44]. Furthermore, the SiO_2_ embedded in the hydrogel matrices releases free Si–O^−^ groups into the SBF. These negatively charged sites attract Ca^2+^ ions electrostatically, forming an activated layer that combines with PO_4_^3−^ to generate Ca_3_(PO_4_)_2_, ultimately transforming into an apatite mineralization layer [45,46]. To further characterize the elemental composition of the mineralized surfaces, energy-dispersive X-ray spectroscopy (EDX) was performed, with the results presented in Figure 6c,d. The Ca/P ratio was found to be 1.59 for the OA/PAAm-GT composite hydrogels, which was below the theoretical value of 1.67. In contrast, the OA/PAAm/SiO_2_-GT composite hydrogels showed a significantly higher Ca/P ratio of 1.95. This elevated value, substantially above the stoichiometric hydroxyapatite ratio of 1.67, suggested the formation of a non-stoichiometric, calcium-rich mineral phase. This deviation could be attributed to the role of SiO_2_, which reacted with water in SBF to generate Si–O^−^ groups. These sites impart a strong negative surface charge, leading to preferential adsorption of Ca^2+^ ions and facilitating the formation of an initial calcium-enriched layer [47]. Such high Ca/P ratios are frequently observed in biomimetic systems and can arise from several factors [45]: the presence of amorphous calcium phosphate (which often exhibits variable composition), the incorporation of other ions (such as CO_3_^2−^ or Mg^2+^) that substitute for phosphate, or the formation of surface-adsorbed Ca-rich complexes prior to crystallization. The measured ratio of 1.95 may indicate a transitional state before the maturation to more stoichiometric apatite, or the formation of a distinct calcium phosphate phase.

### 2.5. In Vitro Cytocompatibility of Composite Hydrogels

**Figure 7:**
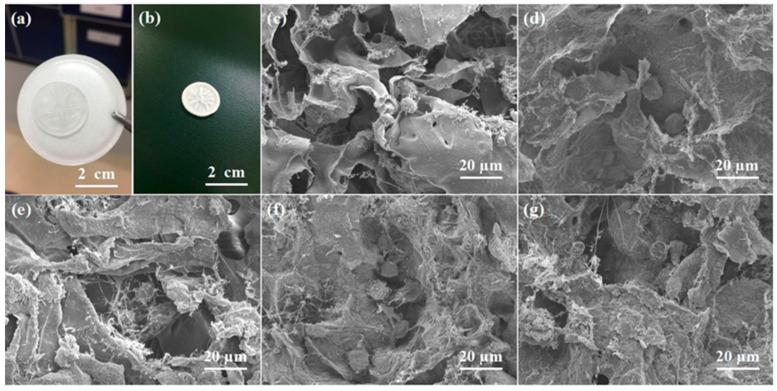
(**a**,**b**) Photographs of OA/PAAm/SiO_2_-GT composite hydrogels for the cytocompatibility measurement; SEM images of MG63 cells cultured on the composite hydrogels for 2 days: (**c**) OA/PAAm-GT; (**d**) OA/PAAm/0.5%SiO_2_-GT; (**e**) OA/PAAm/1.0%SiO_2_-GT; (**f**) OA/PAAm/1.5%SiO_2_-GT; (**g**) OA/PAAm/2.0%SiO_2_-GT.

**Figure 8:**
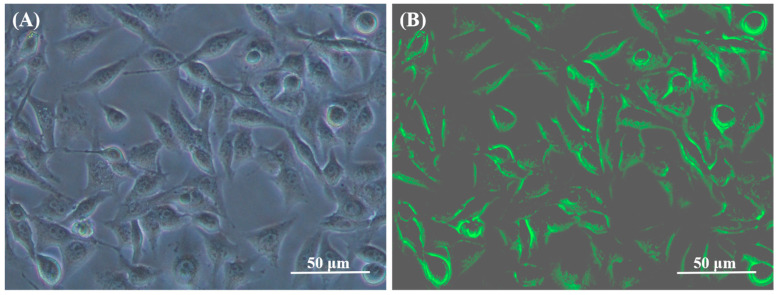
Calcein-AM staining microscopic images of MG63 cells cultured for 48 h on OA/PAAm/1.0%SiO2-GT electrospun composite nanofibers: (**A**) bright field; (**B**) green channel.

To evaluate the cytocompatibility of OA/PAAm/SiO_2_-GT composite hydrogels and investigate the effect of SiO_2_ on cell adhesion, proliferation, and differentiation, MG63 human osteosarcoma cells were seeded and cultured in vitro on the composite hydrogels. Figure 7 illustrates the adhesion of MG63 cells on the composite hydrogels. The cells mainly showed spherical morphology with filamentous extensions and formed clusters on the surface or within the pores of the composite hydrogels. This result implied that the well-defined porous structure of the OA/PAAm/SiO_2_-GT composite hydrogels provided a favorable three-dimensional growth environment for cell proliferation [15,28]. This could be attributed to the composite hydrogel’s ability to mimic the natural extracellular matrix, coupled with the presence of RGD sequences in GT, which were known to promote cell adhesion and proliferation [30,48]. Furthermore, the viability of cells cultured on the OA/PAAm/SiO_2_-GT composite hydrogels was also assessed using Calcein-AM staining. As a live-cell staining probe, Calcein-AM specifically labels viable cells due to the absence of esterase activity in dead cells, making it highly suitable for evaluating cell viability [49]. Figure 8 shows fluorescent images of MG cells stained with Calcein-AM after 48 h of culture on OA/PAAm/SiO_2_-GT composite hydrogels. It was observed that all cells on the OA/PAAm/SiO_2_-GT composite hydrogels were thoroughly stained, indicating that MG cells maintained high viability and were able to survive effectively on these composite hydrogels.

**Figure 9:**
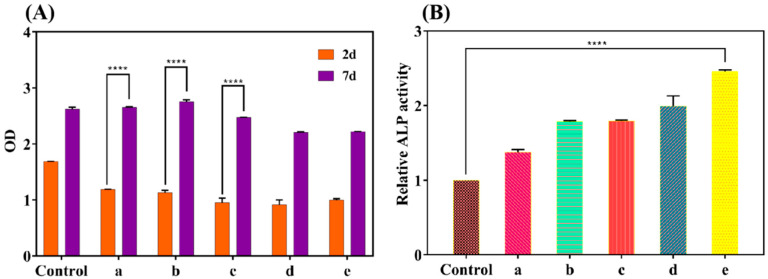
(**A**) Cell proliferation viability of MG63 cells cultured on the composite hydrogels for 2 days and 7 days; (**B**) cell differentiation of MG63 cells cultured on the composite hydrogels for 7 d: (a) OA/PAAm-GT, (b) OA/PAAm/0.5%SiO_2_-GT, (c) OA/PAAm/1.0%SiO_2_-GT, (d) OA/PAAm/1.5%SiO_2_-GT, and (e) OA/PAAm/2.0%SiO_2_-GT (error bars represent the standard deviation of three replicates). **** represents *p* < 0.01, indicating that the difference is statistically significant.

Figure 9A shows the proliferation of MG63 cells on OA/PAAm-GT and OA/PAAm/SiO_2_-GT composite hydrogels. After 2 days of culture, both composite hydrogels showed lower optical density (OD) values than the blank control group. However, after 7 days of culture, a significant increase in cell proliferation was observed in both OA/PAAm-GT and OA/PAAm/SiO_2_-GT composite hydrogels. Notably, the OA/PAAm/0.5%SiO_2_-GT composite hydrogels exhibited the highest OD value, exceeding even the blank control, indicating that an appropriate incorporation of SiO_2_ nanoparticles effectively promoted cell proliferation. This enhancement likely resulted from the negatively charged surface of SiO_2_ nanoparticles, which provided additional binding sites for proteins and thus improved the bioactivity of the composite hydrogels [25]. However, excessive SiO_2_ content reduced the composite hydrogels’ porosity, restricting available space for cell growth and leading to decreased proliferation rates [32,38]. Thus, only an optimal amount of SiO_2_ could effectively support cell growth.

Figure 9B presents the differentiation behavior of MG63 cells on the composite hydrogels, measured by relative alkaline phosphatase (ALP) activity. The OA/PAAm/SiO_2_-GT composite hydrogels showed higher relative ALP activity compared to the blank control, suggesting that SiO_2_ nanoparticles promoted osteogenic differentiation. Furthermore, relative ALP activity increased significantly with higher SiO_2_ content. This effect may be explained by the activation of the extracellular signal-regulated kinase (ERK) pathway in MG63 cells by SiO_2_. Previous studies in animal models have shown that ERK-MAPK activation via tyrosine and threonine phosphorylation stimulates osteoblast differentiation and skeletal development, indicating that SiO_2_ exerted a stimulatory effect on cell differentiation [50,51,52].

## 3. Conclusions

To address the functional limitations of alginate-based hydrogels in biomedical applications, including poor mechanical strength, rapid degradation, and lack of bioactivity, an OA/PAAm/SiO_2_-GT composite hydrogel was developed through an interpenetrating network technique combined with SiO_2_ nanoparticle reinforcement and surface coverage with gelatin. The influence of SiO_2_ content on the microstructure, mechanical properties, swelling behavior, biodegradability, biomineralization capacity, and biocompatibility of the composite hydrogels was systematically investigated. The results demonstrated that SiO_2_ nanoparticles interacted effectively with the polymer matrices. As the SiO_2_ content increased, the porosity of the OA/PAAm/SiO_2_-GT composite hydrogels gradually decreased, while the mechanical properties first improved and then declined. The compressive strength peaked at a SiO_2_ concentration of 1.0% (*w*/*v*). Furthermore, the incorporation of SiO_2_ nanoparticles effectively modulated the in vitro swelling behavior, biodegradation rate, and biomineralization capacity of the composite hydrogels. The OA/PAAm/SiO_2_-GT composite hydrogels also exhibited relatively enhanced cellular adhesion, proliferation, and differentiation, with optimal proliferation observed at a SiO_2_ content of 0.5% (*w*/*v*). Additionally, higher SiO_2_ levels correlated with increased intracellular ALP activity, indicating a promotive effect on osteogenic differentiation of MG63 cells. These findings suggest that incorporating SiO_2_ nanoparticles into OA/PAAm IPN matrices contributes to the enhancement of the biological performance for composite hydrogels, demonstrating their potential applicability in tissue engineering.

## 4. Materials and Methods

### 4.1. Materials and Reagents

Oxidized alginate (OA, *M_W_* = 396,214) with a theoretical oxidation degree of 10% was synthesized according to a previously reported method [26]. Acrylamide (AAm, analytical grade), type A gelatin (GT, biochemical grade), silica nanoparticles (≤50 nm), ammonium persulfate (APS, analytical grade), N,N,N′,N′-tetramethylethylenediamine (TEMED, 99%), N,N′-methylenebisacrylamide (MBA, ≥99%), hydroxyapatite (HAP, biomedical grade), D-glucono-δ-lactone (GDL, 99%) and glutaraldehyde (GA, pharmaceutical grade) were obtained from Aladdin Biochemical Technology Co., Ltd. (Shanghai, China). Human osteosarcoma MG63 cells were procured from the Cell Bank of the Chinese Academy of Sciences (Shanghai, China). DMEM medium, fetal bovine serum, trypsin (0.25% trypsin–EDTA), penicillin, and streptomycin were sourced from HyClone (Thermo Fisher Scientific, Waltham, MA, USA). The Cell Counting Kit-8 (CCK-8) was purchased from Dojindo Laboratories (Kumamoto, Japan). Triton X-100 and the Alkaline Phosphatase (ALP) Assay Kit were acquired from Biyuntian Biotechnology Co., Ltd. (Shanghai, China).

### 4.2. Fabrication of OA/PAAm/SiO_2_-GT Composite Hydrogels

As shown in Scheme 1, OA/PAAm/SiO_2_-GT composite hydrogels were fabricated through a two-step process. First, silica nanoparticles were incorporated into an oxidized alginate/polyacrylamide (OA/PAAm) IPN matrix. Then, the formed composite hydrogel’s surface was covered with gelatin. Specifically, a specified amount of SiO_2_ nanoparticles and 0.37 g of HAP were added to 100 mL of OA solution at 2% (*w*/*v*) concentration, followed by vigorous stirring for 2 h. Subsequently, 4.0 g of AAm, 40 mg of MBA, 40 mg of APS, and 36.4 µL of TEMED were introduced sequentially, and the mixture was stirred for another 20 min. The resulting mixture was poured into a beaker, followed by the addition of 1.29 g of GDL under magnetic stirring. After stirring for 1.0 min, the solution was quickly transferred to a 12-well tissue culture plate (5 mL per well). The sample was first crosslinked at room temperature for 2 h and then further chemically crosslinked in an oven at 65 °C for 5 h. The obtained composite hydrogel was freeze-dried directly. Afterward, the dried sample was immersed in a 1% (*w*/*v*) gelatin (GT) solution for 2 min, followed by overnight treatment in a 2.5% (*w*/*v*) glutaraldehyde (GA) solution. After repeated washing with deionized water, the purified composite hydrogel was freeze-dried again to yield the final OA/PAAm/SiO_2_-GT composite hydrogels. For comparison, samples with silica nanoparticle contents of 0, 0.5% (*w*/*v*), 1.0% (*w*/*v*), 1.5% (*w*/*v*), and 2.0% (*w*/*v*) were labeled as OA/PAAm-GT, OA/PAAm/0.5%SiO_2_-GT, OA/PAAm/1.0%SiO_2_-GT, OA/PAAm/1.5%SiO_2_-GT, and OA/PAAm/2.0%SiO_2_-GT, respectively.

### 4.3. Characterization

The interactions between components in the OA/PAAm/SiO_2_-GT composite hydrogel were characterized by Fourier transform infrared spectroscopy (FT-IR), X-ray powder diffraction (XRD), and thermogravimetric analysis (TGA). FT-IR spectra were acquired using a Nicolet 6700 spectrometer (Thermo Scientific, Waltham, MA, USA) with 64 scans at a resolution of 4 cm^−1^ over the wavenumber range of 4000–400 cm^−1^ to analyze changes in the major functional groups. XRD measurements were performed using an AXS/D8 advance diffract meter (Bruker, Germany) using Cu-Kα (*λ* = 0.154 nm) at 40 kV and 100 mA. The scan range was 2*θ* = 5–60° at a scanning rate of 0.025°/s to determine the crystal structure of the samples. TGA was carried out on a 449F3 thermogravimetric analyzer (Netzsch, Germany) under a N_2_ atmosphere (pressure maintained at 0.04 MPa), with a heating rate of 10 °C/min from 30 to 800 °C, to evaluate the thermal stability of the samples.

Furthermore, the morphology of the OA/PAAm/SiO_2_-GT composite hydrogels was characterized using a JSM-7100F scanning electron microscope (JEOL, Tokyo, Japan). The samples were cut into small cubic pieces, then they were mounted on a copper stub and dried at 40 °C for 6 h. Prior to imaging, the cross-section of the dried sample was sputter-coated with a thin gold layer. The pore structure of the OA/PAAm/SiO_2_-GT composite hydrogels was examined with an AutoPore IV 9500 mercury porosimeter (Micromeritics Instrument Co., Ltd., Shanghai, China). After high-temperature pretreatment, the sample was placed in a sample cell, and pressure was applied to allow mercury to intrude into all pores. Results were processed using built-in microprocessor software (Windows NT V4.00). The mechanical properties of the OA/PAAm/SiO_2_-GT composite hydrogels were tested on a WDW-1 microcomputer-controlled tensile testing machine (Jinan Yinuo Century Testing Instrument Co., Ltd., Jinan, China). The samples were cut into circular disks with a base diameter of 18 mm and a height of 10 mm, and they were compressed perpendicular to their surface at a rate of 5 mm/min until fracture or a strain exceeding 60% was reached. The compressive strength of each sample was determined from the highest point on the linear segment of the obtained stress–strain curve. The compressive strength of each sample was determined from the highest point on the linear segment of the stress–strain curve. The mean value was derived from five independent parallel measurements.

### 4.4. In Vitro Swelling Behavior, Biodegradability, and Biomineralization Measurement

The OA/PAAm/SiO_2_-GT composite hydrogels were immersed in PBS solution and incubated at 37 °C for a period of 2 h. After removal, the surface of the samples was gently wiped with filter paper before weighing. Each experiment was performed in triplicate. The swelling ratio (SR) at each time interval was calculated according to Equation (1), where *W_O_* and *W_S_* denote the mass of the composite hydrogel before and after swelling, respectively.

(1) $$SR=\frac{W_{s}-W_{O}}{W_{O}}\times 100\%$$

Lysozyme (0.01 g) was precisely weighed and placed in a 50 mL beaker. Then, 20 mL of PBS solution was added to prepare a lysozyme-PBS solution with a concentration of 10,000 U/mL. Pre-weighed composite hydrogels (denoted as *W_O_*) were immersed in this solution and degraded in an incubator at 37 °C. Samples were taken out after 7, 14, and 21 days, rinsed gently with deionized water, and dried in an oven at 110 °C until a constant weight was achieved. The weight after drying was recorded as *W_t_*. The biodegradation rate (BR) of the composite hydrogel was evaluated based on weight loss using Equation (2), where *W_O_* and *W_t_* represent the mass of the composite hydrogel before and after biodegradation, respectively.

(2) $$BR=\frac{W_{O}-W_{t}}{W_{O}}\times 100\%$$

Finally, to investigate in vitro biomineralization capability, OA/PAAm/SiO_2_-GT composite hydrogels were immersed in 50 mL of simulated body fluid (SBF) in a beaker for 14 days. Afterwards, the sample was taken out and rinsed repeatedly with deionized water to remove impurities, followed by freeze-drying. The formation of an apatite layer on the composite hydrogel surface was characterized by scanning electron microscopy (SEM), while its elemental distribution was analyzed by energy-dispersive X-ray spectroscopy (EDX).

### 4.5. In Vitro Cytocompatibility Test

The OA/PAAm/SiO_2_-GT composite hydrogels were first sterilized by Cobalt-60 irradiation at a dose of 8 kGy. A 24-well tissue culture plate was used to hold the sterilized materials. Then, 500 µL of DMEM cell culture medium was added to each well and allowed to incubate for 12 h. The biological properties of the samples were evaluated using human osteosarcoma MG63 cells cultured in vitro. MG63 cells at passages 3 to 4 were recovered and allowed to adhere and grow on culture dishes. Then, they were seeded onto the composite hydrogels at a density of 5 × 10^4^ cells per well. As a blank control, cells were seeded in the same manner into a 24-well plate without any material. Additional culture medium was added to bring the total volume in each well to 500 µL. The plates were then incubated at 37 °C in a humidified atmosphere containing 5% CO_2_. The culture medium used throughout the experiments contained 90% DMEM, 10% fetal bovine serum, and 1% penicillin–streptomycin solution. The culture medium was replaced every 2 days.

After 2 days of cell culture on the composite hydrogels, the medium was aspirated from the wells. Then, the samples were gently rinsed several times with PBS. Finally, they were fixed with 2.5% (*w*/*v*) glutaraldehyde solution for 20 min. After fixation, the composite hydrogels were rinsed thoroughly with ultrapure water and subjected to freeze-drying. The morphological characteristics and adhesion behavior of MG63 cells on the composite hydrogel surfaces were examined using SEM. Additionally, the cells adhered to the composite hydrogels for 48 h were washed with PBS, and then they were stained with 5.0 µL Calcein-AM for 20 min. After they were washed with PBS again, the stained cells were visualized using a reconstructive Nikon TieS fluorescence microscope (Nikon, Japan). Cell viability on the composite hydrogels was assessed using a CCK-8 assay kit. After 2 and 7 days of proliferation, the culture medium was replaced, then 50 µL of CCK-8 reagent was added to each well and incubated for 4 h at 37 °C under 5% CO_2_. Then, 100 µL of solution from each well of the 24-well plate was transferred to a 96-well plate, mixed gently, and the optical density (OD) was measured at 450 nm using a Bio-Rad X-mark microplate reader (Hercules, CA, USA).

To evaluate cell osteogenic differentiation, alkaline phosphatase (ALP) activity was measured using a commercial assay kit. The culture medium was supplemented with 0.2 μmol/L dexamethasone and 8.0 mmol/L β-glycerophosphate to promote cell differentiation. After 7 days of culture, the composite hydrogels were washed with PBS and treated with 0.1% (*w*/*v*) Triton X-100 solution for cell lysis under ice-bath conditions. Subsequently, 50 µL of the supernatant was transferred to a 96-well plate, mixed with 50 µL of ALP chromogenic reagent, and incubated for 30 min. The reaction was terminated by adding 100 µL of stop solution, and the OD value was measured at 405 nm using a microplate reader. Control groups, consisting of cells cultured in blank wells without composite hydrogels, were included for comparison in both the cell viability and differentiation assays. All measurements were performed with three independent replicates (*n* = 3). The data are presented as the mean ± standard deviation, and statistical significance was determined by one-way analysis of variance (ANOVA).

### 4.6. Statistical Analysis

OriginPro 8.5 software was used to process and analyze the data, and the results were expressed as mean ± standard deviation. One-way analysis of variance was used for comparison of variables, and *p* < 0.01 indicated that the difference was statistically significant.

## Figures and Tables

**Table 1:** Porosity and pore density of OA/PAAm-GT and OA/PAAm/SiO_2_-GT composite hydrogels with various SiO_2_ content.

Samples	Porosity (%)	Density (mg/mL)
OA/PAAm-GT	88.44	0.133 ± 0.002
OA/PAAm/0.5%SiO_2_-GT	89.35	0.137 ± 0.001
OA/PAAm/1.0%SiO_2_-GT	83.55	0.147 ± 0.002
OA/PAAm/1.5%SiO_2_-GT	80.57	0.154 ± 0.002
OA/PAAm/2.0%SiO_2_-GT	79.64	0.163 ± 0.003

## Data Availability

The raw datasets and data presented in this study are available upon request from the corresponding author.
